# A multiplex PCR assay for rapid identification of major tospovirus vectors reported in India

**DOI:** 10.1186/s12864-020-6560-x

**Published:** 2020-02-18

**Authors:** Sumit Jangra, Anubha Mittal, Heena Dhall, Rakesh Kumar Jain, Amalendu Ghosh

**Affiliations:** Insect Vector Laboratory, Advanced Centre for Plant Virology, ICAR-Indian Agricultural Research Institute, New Delhi, 110012 India

**Keywords:** *Thrips palmi*, *Thrips tabaci*, *Scirtothrips dorsalis*, *Frankliniella schultzei*, Tospovirus transmission, Diagnosis

## Abstract

**Background:**

To date, four thrips vectors have been reported to transmit five different tospoviruses in India. Their identification at an early stage is crucial in formulating appropriate pest management strategies. Since morphometric key-based thrips identification based on the adult stage is time-consuming, there is a need to develop diagnostic tools which are rapid, accurate, and independent of developmental stages. Here, we report a multiplex PCR assay to identify four major thrips vectors viz. *Thrips palmi, T. tabaci, Scirtothrips dorsalis,* and *Frankliniella schultzei* present in India.

**Results:**

Cytochrome oxidase subunit III and internal transcribed spacer region 2 were utilized to design species-specific primers. Of 38 pairs of primers tested, primer pairs AG35F-AG36R, AG47F-AG48R, AG87F-AG88R, and AG79F-AG80R amplified 568 bp, 713 bp, 388 bp, and 200 bp products from the DNA templates of *T. palmi, S. dorsalis, T. tabaci,* and *F. schultzei,* respectively at same PCR conditions. The specificity of the primer pairs was validated with a large number of known specimens and no cross-reactivity was observed with other thrips species. The multiplex PCR assay with a cocktail of all the four primer pairs detected four thrips vectors efficiently and could discriminate all of them concurrently in a single reaction.

**Conclusion:**

The multiplex PCR reported in this study could identify the major thrips vectors reported in India. The assay will be useful in ascertaining distribution profile of major thrips vectors, disease epidemiology, screening large samples, and quarantine.

## Background

The tiny, fringed-winged insects belonging to order Thysanoptera are the sole transmitters of economically damaging tospoviruses (family *Tospoviridae*, order *Bunyavirales*) in ornamental, legume, and vegetable crops. Infection of tospoviruses results in necrotic lesion, stunting, wilting, reduced yield, diminishing quality of produce, and eventually death of plant. Tospoviruses like tomato spotted wilt virus (TSWV) infects more than 900 plant species and impatiens necrotic spot virus (INSV) can be found in over 300 plant species [1]. To date, 16 thrips vectors transmitting more than 29 tospoviruses have been reported [1–4]. Among them, four thrips vectors viz. *Thrips palmi* Karny [5, 6]*, Scirtothrips dorsalis* Hood [7], *T. tabaci* Lindeman [8], and *Frankliniella schultzei* Trybom [7] are reported to transmit five tospoviruses in India [4]. Although *F. occidentalis* (Pergande) has been recently reported in India [9, 10], its distribution and role in spreading tospoviruses under Indian conditions are yet to be studied.

A single thrips species may transmit more than one tospovirus and mixed infestation of more than one thrips vector in a host plant is common. Therefore, identification of thrips vectors at very early stages of infestation is necessary to understand the disease epidemiology and development of appropriate pest management strategies. Since morphometric key-based identification is time consuming, laborious, skill-based, stage-specific, and cannot resolve species ambiguities, there is a need to develop molecular diagnostic techniques that are rapid, specific and independent of developmental stage. PCR-based techniques have proved useful for species-level identification of several thrips species using molecular markers [11–13]. Although cytochrome oxidase subunit I (COI) has been widely used for species level identification of insects, internal transcribed spacer (ITS) and cytochrome oxidase subunit III (COIII) offer additional advantages for identification of thrips at species-level because of larger interspecific distance than COI [14–17, 48]. In the present study, ITS2 and COIII sequences were utilized and a multiplex PCR assay has been successfully developed for simple, rapid, specific, and concurrent identification of major thrips vectors viz. *T. palmi, S. dorsalis, T. tabaci,* and *F. schultzei* present in India.

## Results

### Morphometric and COI-based identification

Based on the morphometric keys, four thrips vector species viz. *T. palmi*, *S. dorsalis*, *T. tabaci*, and *F. schultzei* were identified (Additional file 1: Figure S1) to establish the initial population. Adults of *T. palmi* collected from brinjal plant were pale yellow in color, had seven segmented antennae and three red color ocelli forming a triangle. Interocellar setae did not originate within the ocellar triangle. *S. dorsalis* adults collected from chilli plant had dark wings and dark incomplete stripes on the abdomen. The ocellar setae were between posterior ocelli and forewings had straight cilia. *T. tabaci* adults found on onion were whitish-yellow in color and ocelli were grey with ocellar setae within the ocellar triangle. Adults of *F. schultzei* were identified in the specimens collected from a tomato plant. In microscopic slide of *F. schultzei*, antennae were eight segmented, postocular setae were smaller than interocellar setae located along an imaginary line across the front edges of the two hind ocelli and the posteromarginal comb on abdominal segment VIII was not developed. Further, PCR with primer pair, LCO-1490 and HCO-2198 [18] amplified 560 bp of COI for each of the four thrips vectors. The BLASTn [19] analysis based on 560 bp nucleotide sequences of COI substantiated the morphological identification with > 99% identity.

### Standardization of annealing temperature and PCR conditions

Among the 12 primer pairs designed and tested for *T. palmi* by gradient PCR, only five primer pairs viz. AG35F-AG36R, AG91F-AG92R, AG93F-AG94R, AG95F-A96R, and AG97F-AG98R amplified a product size of 568 bp, 313 bp, 124 bp, 191 bp, and 104 bp, respectively at an annealing temperature ranging from 55 to 65 °C. For *S. dorsalis*, 10 primer pairs were tested of which five primer pairs viz. AG47F-AG48R, AG51F-AG52R, AG53F-AG54R, AG55F-56R, and AG57F-AG58R amplified products of 713 bp, 139 bp, 166 bp, 234 bp, and 218 bp, respectively at a temperature gradient of 55–65 °C for annealing. In case of *T. tabaci,* of the 11 primer pairs tested in gradient PCR with 55–65 °C annealing temperature, five primer pairs viz. AG59F-AG60R, AG67F-AG68R, AG69F-AG70R, AG71F-AG72R, and AG87F-AG88R amplified a product size of 296 bp, 167 bp, 175 bp, 477 bp, and 388 bp, respectively. Only two primer pairs viz. AG75F-AG76R and AG79F-AG80R out of five primer pairs tested could amplify 778 bp and 200 bp products of *F. schultzei* in gradient PCR at the same range of annealing temperature mentioned above.

Based on the results of gradient PCR, 60.4 °C was considered as optimal annealing temperature to proceed multiplex PCR assay. Among the 17 species-specific primer pairs which amplified the respective thrips templates in gradient PCR, five primer pairs (AG91F-AG92R, AG93F-AG94R, AG95F-AG96R for *T. palmi;* AG57F-AG58R for *S. dorsalis;* and AG69F-AG70R for *T. tabaci*) were not taken for further studies as their annealing temperature was either above or below 60.4 °C.

### Cross-reactivity of species-specific primers with other thrips vectors

**Fig. 1:**
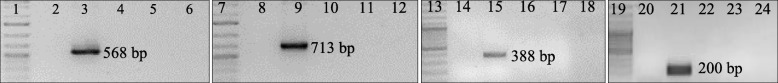
Assessment of cross-reactivity of four species-specific primer pairs. Primer pairs viz. AG35F-AG36R, AG47F-AG48R, AG87F-AG88R, and AG79F-AG80R specific to *T. palmi, S. dorsalis, T. tabaci,* and *F. schultzei*, respectively were shortlisted for multiplex PCR assay. The cross-reactivity of the species-specific primer pairs was assessed in 25 μl PCR reactions and resolved on 0.8% agarose gel electrophoresis. Lane 1, 7: 1 kb plus DNA ladder; Lane 13, 19: 100 bp plus DNA ladder; Lane 2, 8, 14, 20: water control; Lane 3–6: PCR amplicons using *T. palmi*-specific primer pair with DNA templates of *T. palmi* (3), *S. dorsalis* (4*), T. tabaci* (5), and *F. schultzei* (6); Lane 9–12: PCR amplicons using *S. dorsalis*-specific primer pair with DNA templates of *S. dorsalis* (9), *T. palmi* (10), *T. tabaci* (11) and *F. schultzei* (12); Lane 15–18: PCR amplicons using *T. tabaci*-specific primer pair with DNA templates of *T. tabaci* (15), *T. palmi* (16), *S. dorsalis* (17), and *F. schultzei* (18); Lane 21–24: PCR amplicons using *F. schultzei*-specific primer pair with DNA templates of *F. schultzei* (21), *T. palmi* (22), *S. dorsalis* (23), and *T. tabaci* (24). PCR with primer pairs AG35F-AG36R, AG47F-AG48R, AG87F-AG88R, and AG79F-AG80R produced bands of 568 bp, 713 bp, 388 bp, and 200 bp of *T. palmi, S. dorsalis, T. tabaci,* and *F. schultzei*, respectively. No cross-reactivity was found with other thrips vectors

The species-specific primer pairs that showed amplification in the gradient PCR at 60.4 °C were further assessed for cross-reactivity with other thrips vectors. In case of *T. palmi,* two primer pairs (AG35F-AG36R and AG97F-AG98R) showed no cross-reactivity with templates of other vector species. Similarly, no cross-reactivity with other thrips templates was recorded with the primer pairs AG47F-AG48R, AG51F-AG52R, AG53F-AG54R, AG55F-AG56R specific to *S. dorsalis.* The amplicon sizes of the primer pairs, AG97F-AG98R for *T. palmi* and those of AG51F-AG52R, AG53F-AG54R, AG55F-AG56R for *S. dorsalis* ranged from 104 to 234 bp. Considering the size of these amplicons and lack of their resolution in agarose gel electrophoresis, these primer pairs were not considered in multiplex PCR to avoid misinterpretation of the results. Primer pairs AG35F-AG36R and AG47F-AG48R yielding amplicons of 568 bp and 713 bp, respectively were undertaken for concurrent detection of *T. palmi* and *S. dorsalis*. In case of *T. tabaci*, all the primer pairs except primer pair AG87F-AG88R were found cross-reactive with other thrips vectors. Two *F. schultzei*-specific primers (AG75F-AG76R and AG79F-AG80R) amplified products of 788 bp and 200 bp. Keeping in mind the size of other amplicons in triplex PCR described above, primer pair AG75F-AG76R was the first choice for multiplex PCR but found to be cross-reactive with other thrips templates. Hence, the other primer pair for *F. schultzei* (AG79F-AG80R) was tested and no cross-reactivity was recorded. Finally, the four species-specific primer pairs i.e. AG47F-AG48R (derived from COIII), AG35F-AG36R, AG87-AG88R, and AG79F-AG80R (derived from ITS2) which did not show any cross-reactivity with other thrips vectors (Fig. 1) were considered for concurrent detection of four thrips vectors by multiplex PCR.

### Multiplex PCR assay

**Fig. 2:**
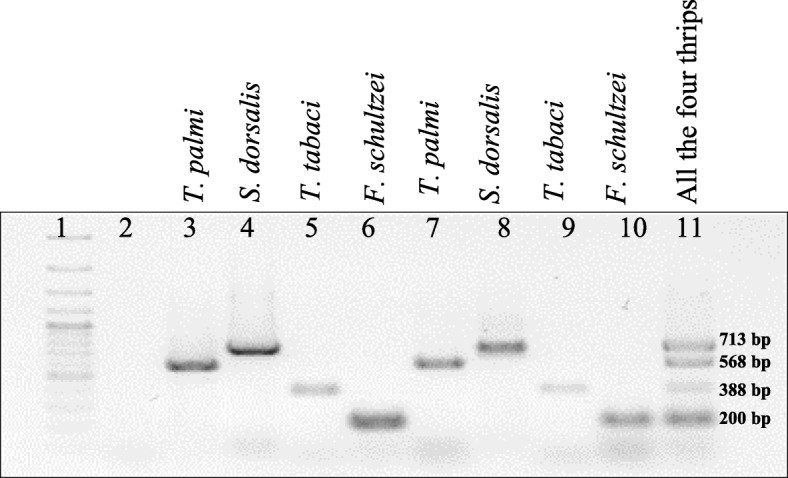
Multiplex PCR assay to identify four thrips vectors concurrently. Multiplex PCR was performed using a cocktail of the four specific-specific primer pairs viz. AG35F-AG36R, AG47F-AG48R, AG87F-AG88R, and AG79F-AG80R for *T. palmi, S. dorsalis, T. tabaci,* and *F. schultzei* with the templates of the four thrips vectors separately and mixed templates of all four thrips vectors. Lane 1: 100 bp plus DNA ladder; Lane 2: water control; Lane 3–6: PCR amplicons using species-specific primers with DNA templates of respective thrips vectors, *T. palmi* (3), *S. dorsalis* (4), *T. tabaci* (5), and *F. schultzei;* Lane 7–11: PCR amplicons using cocktails of primer pairs specific to *T. palmi, S. dorsalis, T. tabaci,* and *F. schultzei* with DNA templates of *T. palmi* (7), *S. dorsalis* (8), *T. tabaci* (9), *F. schultzei* (10), and mixed templates of all four thrips vectors (11). The multiplex PCR using a cocktail of all four thrips vectors amplified products of 568 bp, 713 bp, 388 bp, and 200 bp of *T. palmi, S. dorsalis, T. tabaci,* and *F. schultzei,* respectively. The multiplex PCR efficiently discriminated four thrips vectors individually and even in a single reaction when DNA templates of all four thrips vectors were mixed

The duplex PCR assay performed with a cocktail of AG35F-AG36R and AG47F-AG48R specific to *T. palmi* and *S. dorsalis*, respectively amplified desired products of 568 bp and 713 bp and was able to discriminate between *T. palmi* and *S. dorsalis* (Additional file 2: Figure S2)*.* Triplex PCR using a cocktail of primer pairs AG35F-AG36R, AG47F-AG48R, and AG87F-AG88R amplified 568 bp, 713 bp, and 388 bp products of *T. palmi, S. dorsalis,* and *T. tabaci*, respectively. The triplex PCR was able to discriminate three thrips vectors individually and all of them in a single reaction (Additional file 3: Figure S3). The multiplex PCR to identify all the four thrips vectors viz. *T. palmi, S. dorsalis, T. tabaci,* and *F. schultzei* using a cocktail of AG35F-AG36R, AG47F-AG48R, AG87F-AG88R, and AG79F-AG80R primers yielded products of 568 bp, 713 bp, 388 bp, and 200 bp. The multiplex PCR efficiently discriminated four thrips vectors individually and concurrently even in a single reaction when DNA templates of all four thrips vectors were mixed at a final concentration of 50 ng (Fig. 2). The BLASTn analyses of the sequences of each PCR product further validated the specificity of the reactions with > 98% identity.

### Validation of multiplex PCR assay

**Fig. 3:**
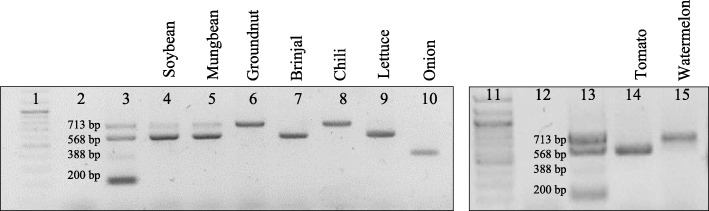
Identification of thrips vectors collected from different crops using multiplex assay. Thrips were collected from brinjal, chilli, onion, lettuce, groundnut, and mungbean plants. Multiplex PCR assay using all four thrips vector-specific primer pairs was performed with DNA templates of thrips collected from different crops. Lane 1, 11: 100 bp plus DNA ladder; Lane 2, 12: water control; Lane 3, 13: multiplex PCR products with mixed templates of all four thrips vectors as positive control; Lane 4–10, 14, 15: multiplex PCR product with template of thrips collected from soybean (4), mungbean (5), groundnut (6), brinjal (7), chilli (8), lettuce (9), onion (10), tomato (14), and watermelon (15). The multiplex PCR assay showed two amplicons of 713 bp and 568 bp with DNA templates of thrips collected from soybean and mungbean plants indicating mixed infestation of *S. dorsalis* and *T. palmi*. A product of 713 bp with template of thrips vectors collected from groundnut, chilli, and watermelon plants confirmed the presence of *S. dorsalis.* Amplification of 568 bp product with thrips templates of brinjal, lettuce, and tomato indicated the presence of *T. palmi*, whereas *T. tabaci* was identified from onion producing 388 bp band

The multiplex PCR was validated over more than 80 reactions with known thrips specimens. The results confirmed the test-retest reliability and reproducibility of the assay. The multiplex PCR assay using the primer cocktail of four species-specific primers pairs (AG35F-AG36R, AG47F-AG48R, AG87-AG88R, and AG79F-AG80R) was employed to identify the thrips vectors in more than 30 collections from natural vegetation. The assay showed two amplicons of 713 bp and 568 bp with DNA templates of thrips collected from soybean and mungbean plants indicating a mixed infestation of *S. dorsalis* and *T. palmi* in these crops (Fig. 3). The multiplex PCR assay with template of thrips vectors collected from groundnut, chilli, and watermelon plants amplified products of 713 bp that confirmed the presence of *S. dorsalis* in groundnut, chilli, and watermelon plants. Similarly, multiplex PCR produced an amplicon of 568 bp and detected the incidence of *T. palmi* on brinjal, lettuce, and tomato plants. The assay confirmed the presence of *T. tabaci* as a 388 bp amplicon was produced when tested with the templates of thrips collected from onion plants. The nucleotide sequence homology analyses of a few representative PCR products showed > 99% identity with the respective thrips vector species that reconfirmed the efficiency of the assay.

## Discussion

Thrips remain major insect pests in vegetables and ornamental plants [20–22]. Besides inflicting direct damages to crops, they transmit deadly tospoviruses. Although the persistent-propagative transmission of tospoviruses by thrips [23] is considered to be specific to virus-vector combination, one thrips vector species can transmit more than one tospovirus [2, 3]. *T. palmi* and *F. occidentalis* are reported to transmit seven different tospoviruses [3, 49]. Transmission of more than one tospovirus is also evident by *F. schultzei, S. dorsalis,* and *T. tabaci* [3]. Infestation of more than one thrips vector in the same host plant has intensified the complexity of the situation. An economic crop like groundnut hosts both *T. palmi* and *S. dorsalis* [24]. More than one thrips vector is also reported in tomato [25], watermelon [26], and soybean [27]. Therefore, development of appropriate methods for fast and concurrent identification of these thrips vectors at an early stage of infestation seems to be essential, especially when they are minute and cryptic in nature and spreading diseases [11, 12, 28]. Morphometric key-based identification of thrips species is time-taking, labor-intensive, requires expertise, and dependent on developmental stage as only adults can be identified. The speed, reproducibility, and accuracy of molecular techniques have made them a valuable tool for identification of thrips vectors [13, 29–36]. The simultaneous identification of several species using multiplex PCR has become popular for its added advantages of saving time, money, and effort over other methods of diagnosis [37, 38].

In India, four thrips vectors viz. *T. palmi, S. dorsalis*, *T. tabaci,* and *F. schultzei* have been reported so far to transmit five tospoviruses such as groundnut bud necrosis virus (GBNV), watermelon bud necrosis virus (WBNV), peanut yellow spot virus (PYSV), capsicum chlorosis virus (CaCV) and Irish yellow spot virus (IYSV) [4]. Recently *F. occidentalis,* a potential vector of TSWV has been reported from the Nilgiri hills of India [9, 10]. Probably, its presence is restricted in that region and role in spreading tospovirus under Indian conditions is yet to be studied. The present study reports a multiplex PCR-based assay for simultaneous identification of four major thrips vectors viz. *T. palmi, S. dorsalis, T. tabaci,* and *F. schultzei* reported in India.

COI is the most extensively used for identification of insect species due to its wide acceptance as a universal barcode [18, 39, 40]. In case of thrips, COI is hyper-variable and considered for haplotype or cryptic species determination [14–17, 48], whereas COIII and ITS regions provide additional advantages at species-level identification due to larger interspecific distance than COI [14, 41]. In the present study, ITS2 and COIII sequences were used to design species-specific primers of the four thrips vectors. Out of 38 species-specific primer pairs designed and tested, four primer pairs derived from ITS2 (AG35F-AG36R, AG87F-AG88R, and AG79F-AG80R) and COIII (AG47F-AG48R) were found suitable to discriminate *T. palmi, T. tabaci, F. schultzei*, and *S. dorsalis* at same PCR conditions. ITS-based markers have been used by various researchers for species-level identification of thrips [11, 12, 29, 42]. The four species-specific primer pairs viz. AG35F-AG36R, AG47F-AG48R, AG87F-AG88R, and AG79F-AG80R for *T. palmi, S. dorsalis, T. tabaci,* and *F. schultzei* used in the present investigation were not cross-reactive and used to carry out concurrent detection of thrips vectors by multiplex PCR. Multiplex PCR has been widely used by various researchers for rapid and simultaneous identification of thrips vectors [28, 34, 38, 41]. However, the present study is unique in the sense that, this is the first effort in concurrent identification of all the major thrips vectors present in India. The multiplex PCR assay reported here was employed to identify thrips vectors collected from the different crops under natural conditions. The assay diagnosed mixed infection of *T. palmi* and *S. dorsalis* in soybean and mungbean plants. *T. palmi* was also identified from the samples collected from brinjal, lettuce, and tomato plants using this assay. The multiplex assay was also found efficient to identify *S. dorsalis* in groundnut, chilli, and watermelon and *T. tabaci* in onion plants.

## Conclusions

The multiplex PCR assay-based technique developed in the present study will be helpful in rapid and simultaneous identification of major thrips vectors transmitting deadly tospoviruses in India. The assay will be helpful in determining the distribution profile of major thrips vectors and understanding disease epidemiology. The assay will facilitate early detection of the thrips vectors to support formulation of suitable management strategies against thrips vectors and tospoviruses. Further, the technique described in the present study can be applied in resistance screening and quarantine.

## Methods

### Sample collection and identification

The initial population of adult thrips was collected in plastic bags from different host plants such as brinjal, chilli, onion, and tomato from the experimental fields of Indian Agricultural Research Institute (IARI), New Delhi (28.6377° N, 77.1571° E, and 228.61 m above MSL) and taken to the laboratory. Microscopic slides were then prepared following the protocol of Silveria and Haro [43] and the thrips vectors were at first identified based on the standard morphometric keys [44, 45]. Morphological characters like antennal segments, color, body shape and size, and position of ocellar setae were taken into consideration. Further, the identity of the initial population of *T. palmi, S. dorsalis, T. tabaci,* and *F. schultzei* was confirmed based on nucleotide sequences of COI. Total DNA from morphologically identified single adult of thrips vectors was extracted using DNeasy Blood and Tissue Kit (Qiagen) following the manufacturer’s protocol. The PCR was carried out as described by Ghosh et al. [46] using the primer pair LCO-1490 and HCO-2198 [18] derived from COI region in a Himedia Prima Duo Thermocycler. The 25 μl PCR mixture contained 20–30 ng DNA, 2.5 μl 10X PCR buffer (Thermo Scientific), 0.4 μM each forward and reverse primer, 260 μM dNTP (Thermo Scientific) and 2 U DreamTaq polymerase (Thermo Scientific). The following PCR conditions were used: 94 °C for 5 min, 35 cycles of 94 °C for 30 s, 54 °C for 1 min 30 s, and 72 °C for 1 min followed by 72 °C for 10 min. The amplified PCR products were eluted, cloned and sequenced to confirm the identity of initial population. The sequences were edited by BioEdit [47] and verified by BLASTn [19] for species homology.

### Development of iso-female lines of thrips vectors

A single adult female of *T. palmi, S. dorsalis, T. tabaci,* and *F. schultzei* identified based on morphometric keys and confirmed by COI nucleotide sequence identity was released on healthy brinjal, chilli, onion, and tomato plants, respectively within insect rearing cages (40 cm * 27 cm * 60 cm) to develop iso-female population of each thrips vector. The plants were grown in plastic pots filled with soilrite and supplied to the thrips. Nutrient solution was provided in a plastic tray on to which the pots were placed and covered by the insect rearing cage. The population was maintained under controlled environmental conditions at 28 **±** 1 °C temperature, 60 **±** 10% relative humidity, and 8 h dark. Fresh plants were provided in the cages as and when required. The population of each thrips vector was regularly monitored for the presence of natural enemies and other thrips species. The purity of the population was checked from time to time by sequencing COI as described above. Adults were collected from the respective iso-female generation with a fine Camel hairbrush for further experiments.

### Designing species-specific primers and standardization of annealing temperature by gradient PCR

For developing species-specific PCR, 38 species-specific primer pairs were designed based on the sequence polymorphism of ITS2 and COIII regions (Additional file 4: Table S1). All known template sequences available in the National Center for Biotechnology Information (NCBI) were utilized for primer designing. The major aspects such as sequence specificity, melting temperature, intra-primer or inter-primer homology were considered for primer designing. Sites with mismatch at 3′-end sequences among the congeneric thrips species were targeted to design the species-specific primers. The site-specificity of the primers was verified by performing BLASTn [19] with available template sequences in NCBI.

Total DNA from respective thrips vectors was extracted as described above. Gradient PCR was performed for each primer pair with DNA template of respective thrips vector to standardize the annealing temperature. The PCR was carried out in 25 μl reaction volume containing ~ 50 ng DNA, 2.5 μl 10X PCR buffer (Thermo Scientific), 0.4 μM each forward and reverse primer, 260 μM dNTPs (Thermo Scientific) and 2 U DreamTaq polymerase (Thermo Scientific). Following reaction conditions were followed, initial denaturation at 94 °C for 5 min, then 35 cycles at 94 °C for 30 s, annealing at a range of 55–65 °C for 45 s, and 72 °C for 30 s followed by a final extension at 72 °C for 10 min. The PCR products were resolved on 0.8% agarose gel stained with GoodView (BR Biochem) and visualized using a gel documentation system (MasteroGen Inc. Taiwan). The optimal annealing temperature for each primer pair was selected based on the resolution of amplified products in agarose gel.

### Cross-reactivity assessment of species-specific primers

Based on the results of gradient PCR, few primer pairs of each thrips vector that could amplify at the same annealing temperature were shortlisted. The shortlisted primer pairs with respect to each thrips vector were assessed for cross-reactivity with DNA templates of other three thrips vectors. To start with the cross-reactivity of *T. palmi*-specific primer pairs, AG35F-AG36R and AG91F-92R were tested with the DNA templates of *S. dorsalis, T. tabaci,* and *F. schultzei* and the cross-reactivity of *S. dorsalis*-specific primer pairs viz. AG47F-AG48R and AG55F-56R was verified with *T. palmi, T. tabaci,* and *F. schultzei* templates. Similarly, PCR was performed with *T. palmi, S. dorsalis,* and *F. schultzei* templates using *T. tabaci*-specific primer pairs viz. AG59F-AG60R, AG71F-AG72R, and AG87F-AG88R. *F. schultzei*-specific primer pairs viz. AG75F-AG76R and AG79F-AG80R were tested with *T. palmi, S. dorsalis,* and *T. tabaci* templates to check the cross-reactivity. PCR was set up in 25 μl reaction mixture and resolved on 0.8% agarose gel electrophoresis as described above. The primer pairs that showed any amplification with DNA templates of other three thrips vectors were not considered in next steps. The primer pairs that were not cross-reactive and the PCR products were well resolved in agarose gel, taken in multiplex PCR assay.

### Multiplex PCR assay for concurrent identification of thrips vectors

Based on the results of gradient PCR, cross-reactivity assay and amplicon sizes, a duplex PCR was performed at first by using a mix of *T. palmi-* and *S. dorsalis*-specific primer pairs viz. AG35F-AG36R and AG47F-AG48R*,* respectively with DNA templates of *T. palmi* and *S. dorsalis* in separate PCR tubes. Same PCR conditions were maintained as described above*.* A triplex PCR assay was performed by using a cocktail of primer pairs viz. AG35F-AG36R, AG47F-AG48R, and AG87F-AG88R specific to *T. palmi, S. dorsalis,* and *T. tabaci,* respectively with templates of *T. palmi, S. dorsalis,* and *T. tabaci* separately at above-mentioned PCR conditions. Further, templates of *T. palmi, S. dorsalis,* and *T. tabaci* were mixed together and 50 ng of the mixed DNA was used in the triplex PCR to test the efficacy of the assay in simultaneous detection of three thrips vectors.

After successful attempts of the triplex PCR, a multiplex PCR was performed which comprised of a cocktail of the four primer pairs viz. AG35F-AG36R, AG47F-AG48R, AG87F-AG88R, and AG79F-AG80R specific to *T. palmi, S. dorsalis, T. tabaci,* and *F. schultzei* in PCR tubes containing the templates of the four thrips vectors separately. The multiplex PCR assay was further tested to detect all the four thrips vectors simultaneously in a single reaction. PCR was conducted using a cocktail of all four species-specific primers described above. DNA templates of all four thrips vectors were mixed to a final concentration of 50 ng keeping other PCR conditions same as mentioned above. Amplified PCR products in the multiplex assay were eluted after agarose gel electrophoresis, cloned, and sequenced to validate the specificity of the reactions. The sequences were processed by BioEdit [47] and species homology was verified by BLASTn [19].

### Validation of multiplex PCR assay

The multiplex PCR assay was validated by using a large number of known specimens. To check the efficiency of the assay in identifying thrips vectors collected from natural vegetation, samples were collected separately from different plants such as brinjal, chilli, onion, lettuce, groundnut, mungbean, tomato, and watermelon from experimental fields of IARI. Thrips were grouped crop-wise and taken to the laboratory. Total DNA was extracted from a group of thrips as described above. Multiplex PCR was performed using the cocktail of all four thrips vector-specific primer pairs with DNA templates of thrips collected from different crops. DNA of four known thrips vectors were mixed and used as positive control and marker. PCR reaction mixture and conditions remained same as mentioned above taking 50 ng DNA template for each reaction. The PCR amplified products were run in 0.8% agarose gel electrophoresis and visualized in a gel documentation system. The sizes of the amplified products were compared with a 100 bp plus DNA ladder (Thermo Scientific) and PCR products of known thrips vectors used as marker. Representative PCR amplicons were cloned and sequenced to revalidate the results.

## Supplementary information

**Additional file 1 Figure S1.** Dorsal view of adult thrips vectors (a) *T. palmi*, (b) *S. dorsalis*, (c) *T. tabaci*, and (d) *F. schultzei*. The microscopic slides were prepared following Silveria and Haro [43]. The thrips vectors were at first identified based on the standard morphometric keys following Bhatti [44], and Cluever and Smith [45] and further confirmed by cytochrome oxidase subunit I sequences.

**Additional file 2 Figure S2.** Duplex PCR to identify *T. palmi* and *S. dorsalis*. Duplex PCR was performed by mixing *T. palmi* and *S. dorsalis*-specific primer pairs viz. AG35F-AG36R, and AG47F-AG48R with DNA templates of *T. palmi* and *S. dorsalis*. Lane 1: 500 bp DNA ladder; Lane 2: water control; Lane 3: PCR amplicon using primer mixer with DNA template of *T. palmi*; Lane 4: PCR amplicon using primer mixer with DNA template of *S. dorsalis*. The duplex PCR assay amplified 568 bp, and 713 bp products of *T. palmi* and *S. dorsalis* and was able to efficiently discriminate between *T. palmi* and *S. dorsalis*.

**Additional file 3 Figure S3.** Triplex PCR assay to identify three thrips vectors concurrently. A triplex PCR assay was performed using a cocktail of primer pairs viz. AG35F-AG36R, AG47F-AG48R, and AG87F-AG88R specific to *T. palmi*, *S. dorsalis*, and *T. tabaci*, respectively with templates of *T. palmi*, *S. dorsalis*, and *T. tabaci* separately and mixed templates of *T. palmi*, *S. dorsalis*, and *T. tabaci*. Lane 1: 100 bp plus DNA ladder; Lane 2: water control; Lane 3–5: PCR amplicons using cocktails of primer pairs specific to *T. palmi*, *S. dorsalis*, and *T. tabaci* with DNA templates of *T. palmi* (3), *S. dorsalis* (4), *T. tabaci* (5), and mixed templates of three thrips vectors (6). Triplex PCR amplified 568 bp, 713 bp, and 388 bp products of *T. palmi*, *S. dorsalis*, and *T. tabaci*, respectively. The triplex PCR was able to discriminate three thrips vectors individually and all of them in a single reaction.

**Additional file 4 Table S1.** Species-specific primer pairs tested for identification of four thrips vectors

## Data Availability

The datasets generated and/or analysed during the current study are available in NCBI database and can be accessed using the accession numbers MN594549, MN194202, MN594551, MN187366, MN193061, MN594550, MN594552, and MT012390.

## Abbreviations

BLAST: Basic local alignment search tool
CaCV: Capsicum chlorosis virus
COI: Cytochrome oxidase subunit I
COIII: Cytochrome oxidase subunit III
GBNV: Groundnut bud necrosis virus
IARI: Indian Agricultural Research Institute
INSV: Impatiens necrotic spot virus
ITS: Internal transcribed spacer
IYSV: Irish yellow spot virus
NCBI: National Center for Biotechnology Information
PYSV: Peanut yellow spot virus
TSWV: Tomato spotted wilt virus
WBNV: Watermelon bud necrosis virus
