# Supplementary material for: A multiplex PCR assay for rapid identification of major tospovirus vectors reported in India
Source: BMC Genomics. 2020 Feb 18;21:170. doi: 10.1186/s12864-020-6560-x (PMC7029577; doi:10.1186/s12864-020-6560-x)

**Supplementary figure 1**


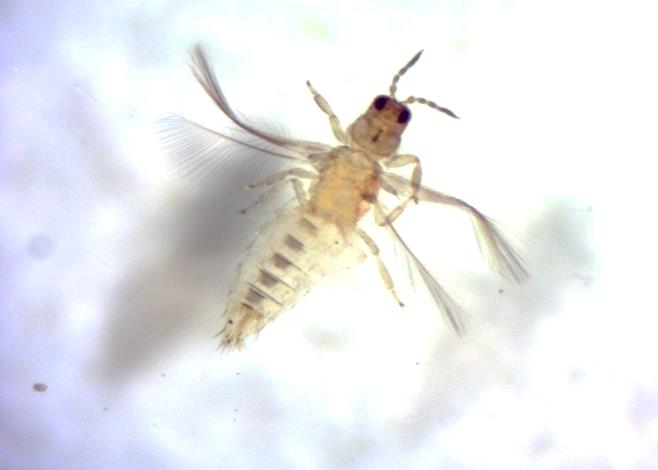

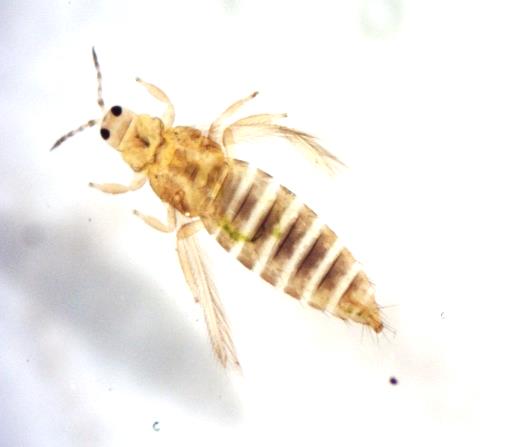

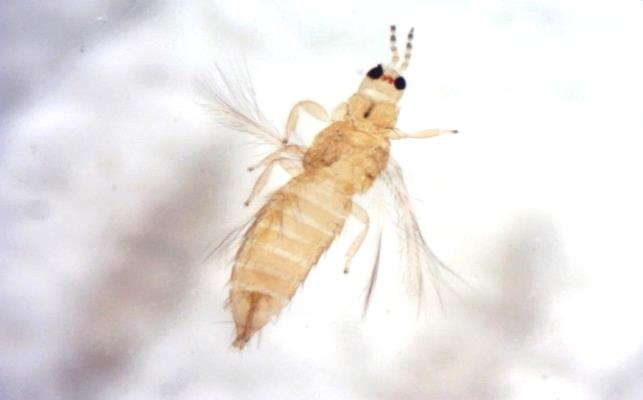


**Dorsal view of adult thrips vectors (a) *T. palmi,* (b) *S. dorsalis,* (c) *T. tabaci,*  and (d) *F. schultzei.*** The microscopic slides were prepared following Silveria and Haro [18]. The thrips vectors were at first identified based on the standard morphometric keys following Bhatti [19], and Cluever and Smith [20] and further confirmed by cytochrome oxidase subunit I sequences.


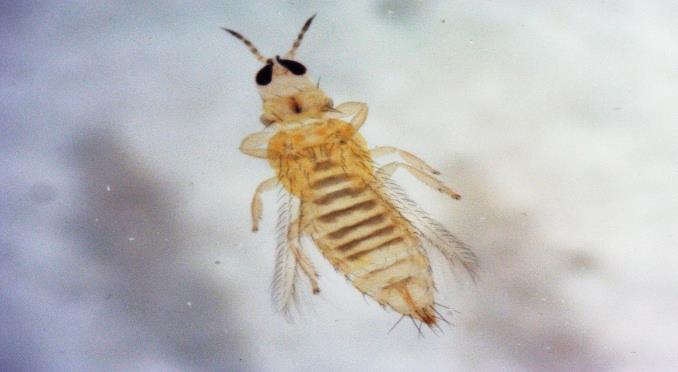

Supplement: Supplementary file 1 — Additional file 1 Figure S1. Dorsal view of adult thrips vectors (a) T. palmi, (b) S. dorsalis, (c) T. tabaci, and (d) F. schultzei. The microscopic slides were prepared following Silveria and Haro [43]. The thrips vectors were at first identified based on the standard morphometric keys following Bhatti [44], and Cluever and Smith [45] and further confirmed by cytochrome oxidase subunit I sequences. [file 12864_2020_6560_MOESM1_ESM.docx]
