# Supplementary material for: A multiplex PCR assay for rapid identification of major tospovirus vectors reported in India
Source: BMC Genomics. 2020 Feb 18;21:170. doi: 10.1186/s12864-020-6560-x (PMC7029577; doi:10.1186/s12864-020-6560-x)
Supplement: Supplementary file 2 — Additional file 2 Figure S2. Duplex PCR to identify T. palmi and S. dorsalis. Duplex PCR was performed by mixing T. palmi and S. dorsalis-specific primer pairs viz. AG35F-AG36R, and AG47F-AG48R with DNA templates of T. palmi and S. dorsalis. Lane 1: 500 bp DNA ladder; Lane 2: water control; Lane 3: PCR amplicon using primer mixer with DNA template of T. palmi; Lane 4: PCR amplicon using primer mixer with DNA template of S. dorsalis. The duplex PCR assay amplified 568 bp, and 713 bp products of T. palmi and S. dorsalis and was able to efficiently discriminate between T. palmi and S. dorsalis. [file 12864_2020_6560_MOESM2_ESM.docx]

**Supplementary figure 2**


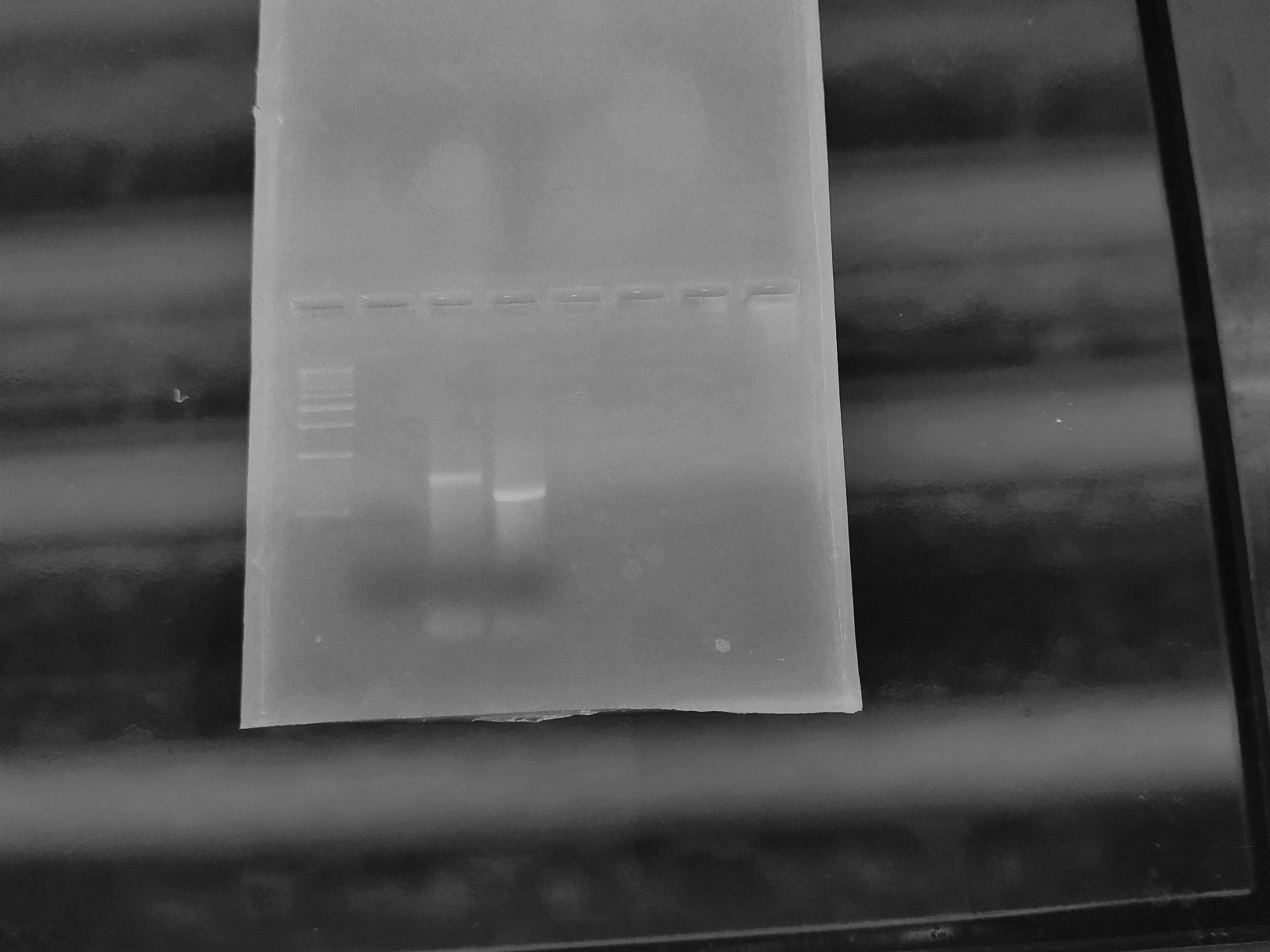


1 2 3 4

**713 bp**

**568 bp**

**Duplex PCR to identify *T. palmi* and *S. dorsalis***

Duplex PCR was performed by mixing *T. palmi* and *S. dorsalis* specific primer pairs *viz*. AG35F-AG36R, and AG47F-AG48R with DNA templates of *T. palmi* and *S. dorsalis.* Lane 1: 500 bp DNA ladder; Lane 2: water control; Lane 3: PCR amplicon using primer mixer with DNA template of *T. palmi;* Lane 4: PCR amplicon using primer mixer with DNA template of *S. dorsalis*. The duplex PCR assay amplified 568 bp, and 713 bp products of *T. palmi* and *S. dorsalis* and was able to efficiently discriminate between *T. palmi* and *S. dorsalis.*
