# Supplementary material for: A multiplex PCR assay for rapid identification of major tospovirus vectors reported in India
Source: BMC Genomics. 2020 Feb 18;21:170. doi: 10.1186/s12864-020-6560-x (PMC7029577; doi:10.1186/s12864-020-6560-x)
Supplement: Supplementary file 3 — Additional file 3 Figure S3. Triplex PCR assay to identify three thrips vectors concurrently. A triplex PCR assay was performed using a cocktail of primer pairs viz. AG35F-AG36R, AG47F-AG48R, and AG87F-AG88R specific to T. palmi, S. dorsalis, and T. tabaci, respectively with templates of T. palmi, S. dorsalis, and T. tabaci separately and mixed templates of T. palmi, S. dorsalis, and T. tabaci. Lane 1: 100 bp plus DNA ladder; Lane 2: water control; Lane 3–5: PCR amplicons using cocktails of primer pairs specific to T. palmi, S. dorsalis, and T. tabaci with DNA templates of T. palmi (3), S. dorsalis (4), T. tabaci (5), and mixed templates of three thrips vectors (6). Triplex PCR amplified 568 bp, 713 bp, and 388 bp products of T. palmi, S. dorsalis, and T. tabaci, respectively. The triplex PCR was able to discriminate three thrips vectors individually and all of them in a single reaction. [file 12864_2020_6560_MOESM3_ESM.docx]

**Supplementary figure 3**


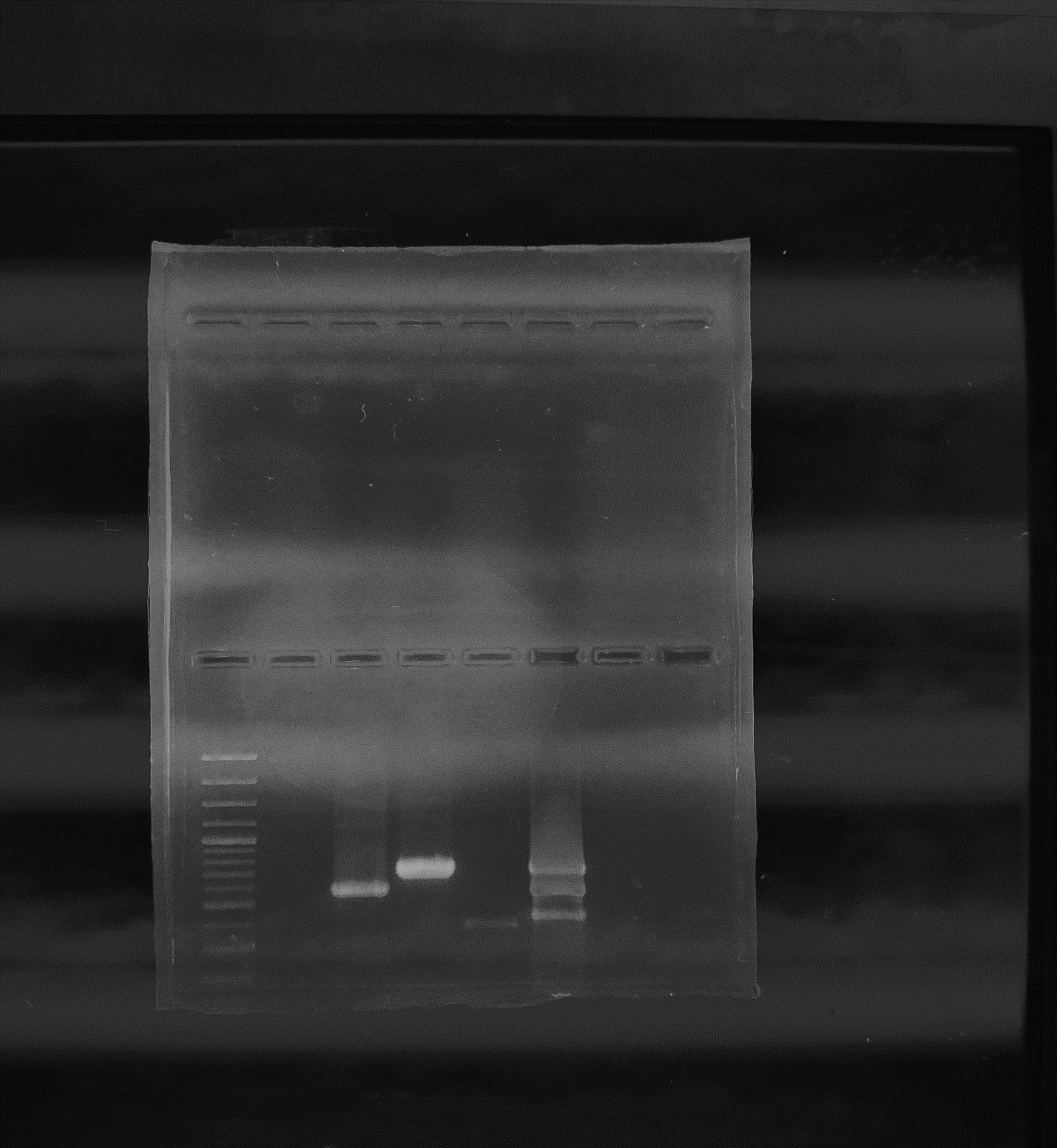


1 2 3 4 5 6 7

**713 bp**

**568 bp**

**388 bp**

**Triplex PCR assay to identify three thrips vectors concurrently**

A triplex PCR assay was performed using a cocktail of primer pairs *viz*. AG35F-AG36R, AG47F-AG48R, and AG87F-AG88R specific to *T. palmi,* *S. dorsalis,* and *T. tabaci,* respectively with templates of *T. palmi,* *S. dorsalis,* and *T. tabaci* separately and mixed templates of *T. palmi,* *S. dorsalis,* and *T. tabaci*. Lane 1: 100 bp plus DNA ladder; Lane 2: water control; Lane 3-5: PCR amplicons using cocktails of primer pairs specific to *T. palmi,* *S. dorsalis,* and *T. tabaci* with DNA templates of *T. palmi* (3), *S. dorsalis* (4), *T. tabaci* (5), and mixed templates of three thrips vectors (6). Triplex PCR amplified 568 bp, 713 bp, and 388 bp products of *T. palmi, S. dorsalis,* and *T. tabaci*, respectively. The triplex PCR was able to discriminate three thrips vectors individually and all of them in a single reaction.
