# Supplementary material for: A multiplex PCR assay for rapid identification of major tospovirus vectors reported in India
Source: BMC Genomics. 2020 Feb 18;21:170. doi: 10.1186/s12864-020-6560-x (PMC7029577; doi:10.1186/s12864-020-6560-x)
Supplement: Supplementary file 4 — Additional file 4 Table S1. Species-specific primer pairs tested for identification of four thrips vectors [file 12864_2020_6560_MOESM4_ESM.docx]

**Supplementary table 1 Species-specific primer pairs tested for identification of four thrips vectors**

| **Sl. No.** | **Thrips vector** | **Forward Primer (5′→3′)** | | **Reverse Primer** **(5′→3′)** | | **Amplicon size (bp)** | **Genomic region** |
| --- | --- | --- | --- | --- | --- | --- | --- |
| 1. | *T. palmi* | AG23 | AG35TAACAGCAGCTCATCATT | AG24 | AATTGAGTCTGCGATTGT | 110 | COIII |
| 2. |  | AG25 | GATCAGGAGTGACATTAACAG | AG26 | GACCCATAAATTGAGTCTGC | 128 | COIII |
| 3. |  | AG27 | ACACGCTTGGCGTGGATG | AG28 | ACCGGCGGTGTCAAGAAG | 221 | H3 |
| 4. |  | AG29 | ACGAGGTAGGCCTCGGAA | AG30 | CGCTCTTCGTGAGATCCG | 129 | H3 |
| 5. |  | AG31 | AACGTACCAAAACTCTACGAGTACG | AG32 | GCCGCAGTCCACGAACAA | 766 | ITS1 |
| 6. |  | AG33 | GCTCGCCTTCCTAACCAGTC | AG34 | GGAACATTAGCCGTGCTTCG | 666 | ITS1 |
| 7. |  | **AG35** | **GTGAACTGCAGGACACAT** | **AG36** | **CACCTGAACAGAGGTCGG** | **568** | **ITS2** |
| 8. |  | AG37 | TGGCTGCTGAACCGCTCC | AG38 | GGTGCCCACTCTGTCCTG | 175 | ITS2 |
| 9. |  | AG91 | GTTTATATAAAGAAGGAGC | AG92 | GATGTTGATAAAGTACAG | 313 | COI |
| 10. |  | AG93 | CTCTTTTAATTATAGGTTTATATAAAG | AG94 | TTTAAAATTGTAGTAATGAAATT | 124 | COI |
| 11. |  | AG95 | TATAGTTCCCGACAATGA | AG96 | TATAGACCCGGTTTAGTAA | 191 | COII |
| 12. |  | AG97 | CAATGTTTAACTACCTCTCTAG | AG98 | TTCTGCGCATTGACCATA | 104 | COIII |
| 13. | *S. dorsalis* | AG39 | GGCATTTTATATTGTATATTTGG | AG40 | CCTCCTGGATCAAAAAATG | 580 | COI |
| 14. |  | AG41 | GATCTGGTATACTTGGATTATCAC | AG42 | GTAATAGCACCCGCTAAAACAG | 498 | COI |
| 15. |  | AG43 | GACATCGTTTTTAGCAAAATTTC | AG44 | GCTTCAATACAAATAGGTATAAATGC | 620 | COII |
| 16 |  | AG45 | GCCTATCCTTCTCTTTACTACC | AG46 | CTGTTCCACAAATTTCTGCA | 355 | COII |
| 17. |  | **AG47** | **GTTCCATTTCATTTAGTTTCACC** | **AG48** | **GTCATACTACGTCAACAAAATGTC** | **713** | **COIII** |
| 18. |  | AG49 | CAGCTAAAAGATTTAATATAAAAGC | AG50 | CGCAGATTCAGTATATGGTTCAAC | 450 | COIII |
| 19. |  | AG51 | TGGAGTTCCGTGCTCTTC | AG52 | CAGATCTTCACTGTCAACTAC | 139 | htpG |
| 20. |  | AG53 | GTGAAGCACTTCTCAGTTG | AG54 | CAGATCTTCACTGTCAACTAC | 166 | htpG |
| 21. |  | AG55 | GGACGAGATTGGAGTCTC | AG56 | CCTGGATCTTTGTCCCGA | 234 | ITS2 |
| 22. |  | AG57 | GGACGAGATTGGAGTCTC | AG58 | CTTGAGCACAGTGGTCCC | 218 | ITS2 |
| 23. | *T. tabaci* | AG59 | CTGACATAGCATTCCCTCG | AG60 | CGCTAACACTGGCAAAGAT | 296 | COI |
| 24. |  | AG61 | CGATTAAATAATATAAGATTCTGAC | AG62 | GAAGAATGGCTGTTAAAATAAC | 245 | COI |
| 25. |  | AG63 | AATTCCAGACAGAGAAATAC | AG64 | GTCCATAATAGATTCCCGG | 194 | COII |
| 26. |  | AG65 | CCAGACAGAGAAATACTTAAAC | AG66 | GTCCATAATAGATTCCCGG | 188 | COII |
| 27. |  | AG67 | GCATCAGAAGTGACTTTAAC | AG68 | GAATTCCGTGAAATCCTG | 167 | COIII |
| 28. |  | AG69 | ATTTATTGCATCAGAAGTG | AG70 | GAATTCCGTGAAATCCTG | 175 | COIII |
| 29. |  | AG71 | CCTCGGTCTTTGCACCTT | AG72 | TGAACTGAGGTCGGATTACAA | 477 | IST2 |
| 30. |  | AG73 | CCACTCCTGGCTGAGGGT | AG74 | ACATTCCACTCAGGCGCA | 379 | ITS2 |
| 31. |  | AG85 | CAGACTGCCCAAGCGAC | AG86 | AGTGATGCAGCACAACAC | 356 | ITS2 |
| 32. |  | **AG87** | **GGCCCTCGGTCTTTGCA** | **AG88** | **TCAGGCGCACGCTAAATGCA** | **388** | **ITS2** |
| 33. |  | AG89 | AGCGACAGCACACACCTC | AG90 | CTGGGGTCTTCACGCCC | 269 | ITS2 |
| 34. | *F. schultzei* | AG75 | GGTGTCGTCAGCTCGTGC | AG76 | GAAGCAAGCCGGAGTTCTC | 778 | 28s rRNA |
| 35. |  | AG77 | GTTAGAGATGGTGTCGTC | AG78 | GAACCATCTAGTAGCTGGTTC | 646 | 28s rRNA |
| 36. |  | **AG79** | **TCGTAACAAGGTTTCCGTAG** | **AG80** | **CAATGTGCGTTCGAAATG** | **200** | **ITS2** |
| 37. |  | AG81 | GTAGGTGAACCTGCGGAAG | AG82 | CGGACCGTCGAATGAATAATC | 688 | ITS2 |
| 38. |  | AG83 | GGAGCATTAAATTTTATTACTACC | AG84 | GATATAAAATTGGGTCTCCAC | 187 | COI |
